# MKS1 interacts with components of the ubiquitin-proteasome pathway to regulate ciliogenesis and multiple signalling pathways

**DOI:** 10.1186/2046-2530-1-S1-P108

**Published:** 2012-11-16

**Authors:** G Wheway, Z Abdelhamed, S Natarajan, CA Johnson

**Affiliations:** 1 Leeds Institute of Molecular Medicine, UK

## 

MKS1, a ciliary protein containing a B9 domain of unknown function, plays an important role in ciliogenesis. Mutation of the MKS1 gene causes the neonatal lethal multi-organ developmental condition Meckel-Gruber syndrome, characterized by severe ciliary defects and disruption of both Wnt and Shh signalling. We have performed a yeast two-hybrid screen for the MKS1 B9 domain and identified and validated interactions between MKS1, and both an E2 ubiquitin conjugating enzyme and an E3 ubiqutin ligase. Previous studies have shown the importance of the basal body in regulating Wnt signalling through selective proteolysis and the study of the MKS1 protein offers additional mechanistic insight into this process. We present evidence that the role of MKS1 in ciliogenesis and developmental signalling is mediated by targeted protein degradation. Work on a newly characterised *Mks1* mutant mouse also provides further insight into the role of this particular ciliary protein normal processes of in vivo developmental signalling regulation and its disruption in Meckel-Gruber syndrome.

